# Challenging the *Metallothionein* (*MT*) Gene of *Biomphalaria glabrata*: Unexpected Response Patterns Due to Cadmium Exposure and Temperature Stress

**DOI:** 10.3390/ijms18081747

**Published:** 2017-08-11

**Authors:** Michael Niederwanger, Martin Dvorak, Raimund Schnegg, Veronika Pedrini-Martha, Katharina Bacher, Massimo Bidoli, Reinhard Dallinger

**Affiliations:** Institute of Zoology and Center of Molecular Biosciences Innsbruck (CMBI), University of Innsbruck, Technikerstrasse 25, A-6020 Innsbruck, Austria; michael.niederwanger@uibk.ac.at (M.N.); martin.dvorak@uibk.ac.at (M.D.); raimund.schnegg@uibk.ac.at (R.S.); Veronika.Pedrini-Martha@uibk.ac.at (V.P.-M.); csap9255@student.uibk.ac.at (K.B.); massimo.bidoli@student.uibk.ac.at (M.B.)

**Keywords:** metallothionein, metal binding domain, cadmium tolerance, heat shock, cold shock, *Biomphalaria glabrata*, *Gastropoda*, *Hygrophila*

## Abstract

Metallothioneins (MTs) are low-molecular-mass, cysteine-rich, metal binding proteins. In most animal species, they are involved in metal homeostasis and detoxification, and provide protection from oxidative stress. Gastropod MTs are highly diversified, exhibiting unique features and adaptations like metal specificity and multiplications of their metal binding domains. Here, we show that the MT gene of *Biomphalaria glabrata*, one of the largest *MT* genes identified so far, is composed in a unique way. The encoding for an MT protein has a three-domain structure and a C-terminal, Cys-rich extension. Using a bioinformatic approach involving structural and in silico analysis of putative transcription factor binding sites (TFBs), we found that this *MT* gene consists of five exons and four introns. It exhibits a regulatory promoter region containing three metal-responsive elements (MREs) and several TFBs with putative involvement in environmental stress response, and regulation of gene expression. Quantitative real-time polymerase chain reaction (qRT-PCR) data indicate that the *MT* gene is not inducible by cadmium (Cd) nor by temperature challenges (heat and cold), despite significant Cd uptake within the midgut gland and the high Cd tolerance of metal-exposed snails.

## 1. Introduction

The adaptation process of animal species towards stressful conditions is achieved, among other methods, by relying on an array of “stress”-related gene encoding for proteins enabling protection against adverse environmental conditions, repair of damaged macromolecules, and detoxification of unwanted metal ions. Metallothioneins (MTs), in particular, belong to a superfamily of nonenzymatic metal binding proteins, occurring in bacteria and most eukaryotic kingdoms [1,2]. Typically, MTs are hydrophilic, low-molecular-mass proteins binding with high affinity to a number of transition metal ions such as Cd^2+^, Zn^2+^ and Cu^+^ [3,4,5]. One of their most distinctive properties is the high cysteine content (up to ~30%) with individual cysteine residues arranged in distinctive motifs (e.g., Cys-x-Cys, Cys-x-x-Cys) that form metal-binding clusters. Owing to their features, MTs are often involved in the homeostatic regulation and detoxification of metallic trace elements [6,7]. Moreover, MTs may also participate in the cellular protection from oxidative stress [8,9].

Gastropods thrive in marine, terrestrial and freshwater habitats and possess MTs with striking capabilities for protecting their hosts from metal stress [10,11,12,13,14] and adverse environmental conditions [15]. In fact, the primary structure of MTs from species of different gastropod clades is highly variable, suggesting a huge potential for evolutionary adaptation to different habitats and their varying environmental challenges [12,16].

Surprisingly, there is little knowledge available about the role and biological function of MTs in freshwater pulmonate snails [17,18], many of them belonging to the clade of *Hygrophila*. An important member of *Hygrophila* is *Biomphalaria glabrata*. This freshwater snail has attained a particular significance as an intermediary host of the trematode parasite *Schistosoma mansonii*, which leads to infections with schistosomiasis of millions of people worldwide every year [19]. Like many other species of *Hygropohila*, *Biomphalaria glabrata* can be considered as a (phylogenetically spoken) modern freshwater snail with a “lung”, consisting of the mantle cavity. Species of this group carry an air bubble within this cavity from which oxygen enters the blood vessels directly by diffusion. This feature enables aquatic lung snails to remain under water over extended periods of time. The adaptive transition from marine to freshwater conditions may have evolved through a number of preadaptations in marine ancestors [20], confronting freshwater neocolonizers with the necessity to readjust their mechanisms for energy acquisition and osmotic regulation [21,22,23,24]. This may also have implications on their mechanisms for metal uptake and regulation [25,26].

Here we report the identification and annotation of an *MT* gene locus from the ram’s horn snail, *Biomphalaria glabrata*, provide an analysis of its gene structure and promoter region in comparison with other gastropod *MT* genes, identify allelic variation and assess mRNA transcription in response to Cd and nonmetallic stressors.

## 2. Results and Discussion

### 2.1. Gene Map and Coding Sequence

**Figure 1:**
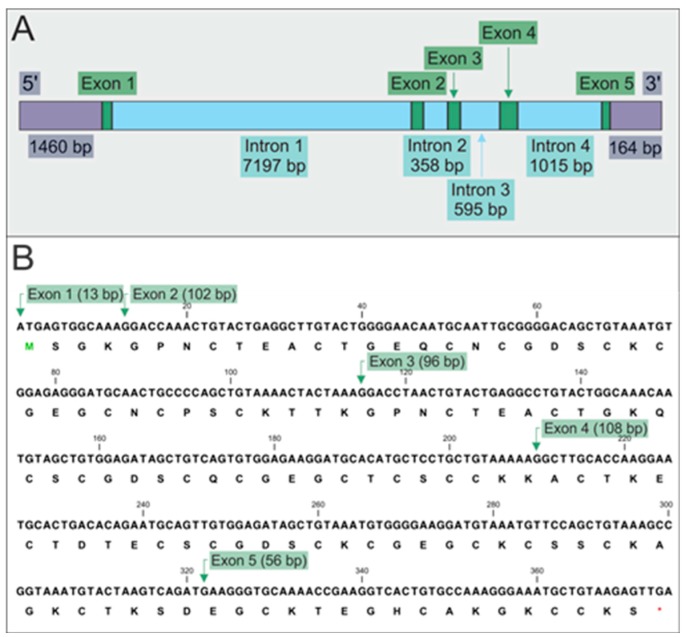
Structural organization of the *Metallothionein* (*MT*) gene from *Biomphalaria glabrata*. (**A**) Map showing the exon/intron structure of the gene. Positions and length of exons (green), introns (blue), the promoter at the 5′ end-and the 3′-untranslated region (UTR) (lilac) are specified by boxes above and underneath the gene map; (**B**) linked exons 1–5 (shaded in green) with starting points (green arrows) and length (in parentheses), coding for amino acid sequence of the MT protein (shown in one-letter amino acid code below base triplets).

The *MT* gene of *Biomphalaria glabrata* is more than 11,000 bp long. It consists of five exons and four introns with differing lengths (Figure 1A), the promoter region and the 3′ untranslated region. The translated coding region represented by the five exons (Figure 1B) spans 369 bp and was confirmed by sequencing of polymerase chain reaction (PCR)-amplified and cloned individual samples. Analysis of the gene by the software package of the transcription factor database (TRANSFAC) yielded a large number of putative transcription factor binding sites (TFBs), related to stress response and regulatory functions, interspersed throughout the whole gene sequence with theoretical binding sites for the TATA-binding protein (TBP), Cdx homeodomeain protein 1 (Cdx-1), MADS box transcription enhancer factor 2 (MEF2C), yeast activator protein 1 (YAP-1) and heat shock factors (HSFs) (for functional explanation, see Table 1) being the most abundant.

### 2.2. Promoter Structure and Metal-Responsive Elements

**Figure 2:**
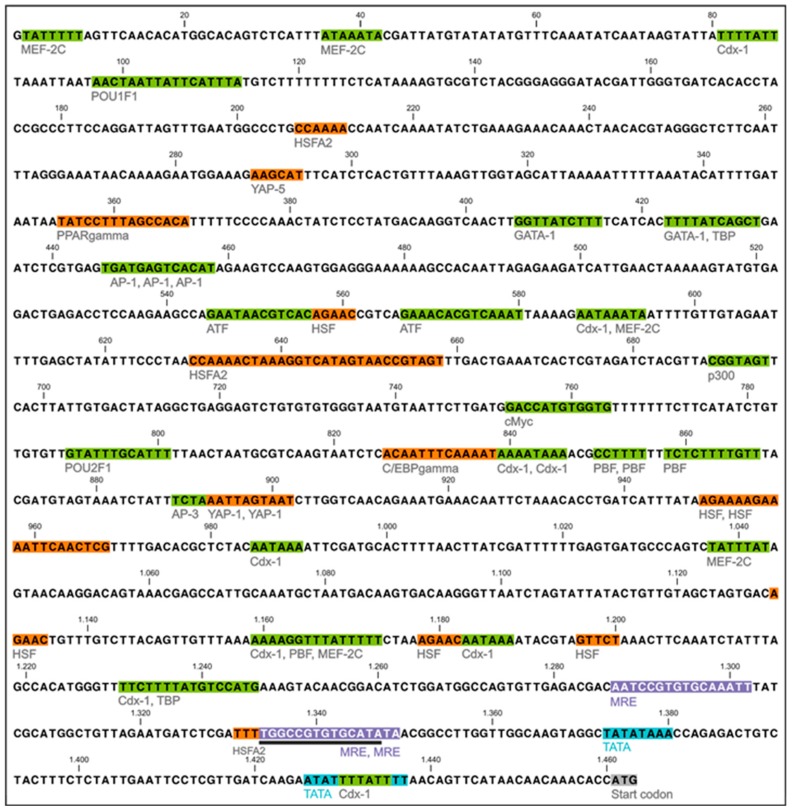
Nucleotide sequence of the promoter region (position 1 to 1460 bp upstream from the start codon), of the *MT* gene of *Biomphalaria glabrata*. Metal responsive elements (MREs) acting as potential binding sites for the presumed metal transcription factor I are highlighted in lilac. Potential transcription factor binding sites (TFBs) involved in stress response are marked in orange and hypothetical binding sites for regulatory transcription factors are marked in green. The putative TATA boxes are highlighted in blue and the start codon of the first exon is shaded in grey. Functional specification of TFBs identified using TRANSFAC: metal induction (lilac): MREs: aatccgtGTGCAaatt, tggccgtGTGCAtata, tggccgtGTGCAta (black line indicates palindromic MRE sequence of the MRE in opposite direction). Stress response (orange): HSF: GTTCT, AGAAC, agaaaattcaACTCG, AGAAAagaaaattca, TGAAGagaatatgcg; C/EBPγ: acaATTTCaaaat; PPARγ: tatCCTTTagccaca; YAP: aTTAGTaa, aAGCAT, agcaggATACGtaatgtatt. Transcriptional regulation (green): AP-1: atgAGTCAc, tgaTGAGTcacat; ATF: gaaacaCGTCAa, gaataaCGTCAc; Cdx-1: TTTATt, ttctTTTATgtccatg, TTTATt, aATAAA; cMyc: gaccATGTGgtg; GATA-1: ttTTATCagc, ttTTATCtct, gccctTATCAtttt; MEF-2C: atAAATA, TATTTtt; POU2F1: aTTTGCatt; POU1F1a: aattATTCAt; p300: cgGTAGT, ACGTTcg; PBF: tctCTTTTgtt, CCTTTt; TBP: tTTTATgtcca, tTTTATcagct. For abbreviations and functional explanation of the single TFBs, see Table 1.

**Figure 3:**
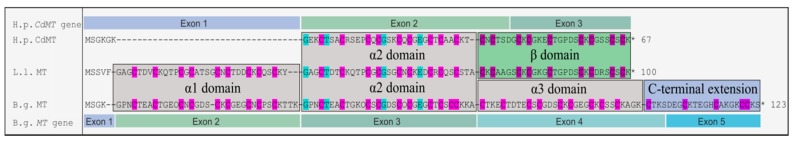
Alignment of the CdMT of *Helix pomatia* (H.p.) (GenBank acc. Nr.: AF399740.1), the MT of *Littorina littorea* (L.l.) (GenBank acc. Nr.: AY034179.1) and the MT of *Biomphalaria glabrata* (B.G.) (GenBank acc. Nr.: KT697617) displaying the amino acid sequence and suggested domain structure. Exon structures of the genes are shown for *Helix pomatia* (H.p. *CdMT* gene) (GenBank acc. Nr.: FJ755002.1) and for *Biomphalaria glabrata* (B.g. *MT* gene) (GenBank acc. Nr.: XM_013225031). Conserved cysteines are marked in pink. Conserved amino acid positions shared by all MTs are marked in blue. The α-domains are indicated by a grey box, β-domains by a green box and the C-terminal extension is marked by a blue box. Exon structures of the genes are indicated by colored boxes above (for H.p.) and underneath (for B.g.) the respective amino acid sequence. Additionally, the length is indicated at the end of each sequence.

Referring to the promoter analysis of the *CdMT* gene of *Helix pomatia* [16] and due to the abundance of putative Transcription Factor Binding Sites (TFBs), the promoter region of the *MT* gene of *Biomphalaria glabrata* was deliberately restricted to a length of 1460 bp (Figure 2). An analysis of this promoter region by the software package TRANSFAC indicated the presence of a large number of putative TFBs involved in stress response or transcriptional regulation (mostly with activating and enhancing functions) (Table 1). Expectedly, the promoter also contains three metal-responsive elements (MREs), considered to be the most characteristic TFBs of an *MT* gene. They are located in the proximal promoter region upstream from the start codon at positions −173 and −127 bp. Two of them consist of a bidirectional, palindromic sequence at −127 bp. No TATA box motif was detected by TRANSFAC. However, two sequence stretches were identified by us as potential TATA box-like motifs, located at positions −24 bp and −80 bp upstream of the start codon (Figure 2). Compared to the already characterized *MT* gene of *Helix pomatia* [16], the *MT* promoter of *Biomphalaria glabrata* differs from the former by the kind, number and allocation of putative TFBs. Moreover, the respective encoded MT protein shows a different number and organization of metal-binding domains, with different positions of cysteine residues within the primary structure compared to the *CdMT* of *Helix pomatia*, which suggesting specific adaptation strategies in the two species towards metal challenge and environmental stressors. In particular, the *Biomphalaria glabrata MT* gene promoter contains a proximal cluster of three MREs and in close vicinity to them also hosts putative binding sites for transcriptional activators and enhancers (Cdx-1, MEF-2C, prolamin box binding factor (PBF), and TBP) (Figure 3, Table 1).

In contrast, the *CdMT* gene promoter of *Helix pomatia* contains four MREs, one of them at a distal position [16]. As in the case of *Helix pomatia*, the *MT* gene promoter of *Biomphalaria glabrata* also exhibits several theoretical TFBs for transcription factors involved in stress response and stress-related signaling (C/enhancer binding protein (C/EBPβ), heat shock element (HSE), peroxisome proliferator activated receptor (PPAR), and yeast activator protein (YAP)). A prominent feature of the *Biomphalaria glabrata MT* gene promoter is, moreover, its preponderant abundance of putative HSEs acting as binding sites for HSFs, suggesting that the *MT* gene of this species may perhaps be upregulated not only by metal stress, but also by nonmetallic stressors like heat shock. In this regard, it has been shown that some *MT* genes can be directly induced by stress-related transcription factors, including the heat shock transcription factor [27]. In particular, some *MT* genes may be activated by nonmetallic inducers, often through synergistic interaction of MREs with other stress-related TFBs such as antioxidant-responsive elements upon oxidative stress [28]. Overall, transcriptional upregulation by nonmetallic stressors like hypoxia, oxidative stress, physical stress and starvation, among others, is a common feature of many *MT* genes [16,29,30,31,32]. Interestingly, the expression of the heat shock protein 70 (HSP70) in *Biomphalaria glabrata* can be influenced by Cd exposure [33]. Our present study shows, however (see below), that the *MT* gene itself does not or only hesitantly react towards temperature-associated stress.

### 2.3. Domain Structure of the MT Protein

The translated primary structure suggests that the MT protein of *Biomphalaria glabrata* is a three-domain MT with three α domains and an additional, cysteine-containing C-terminal extension (Figure 1B and Figure 3) [35]. The first N-terminal (α1) and the third C-terminal α-domain (α3) contain nine cysteine residues each, whereas the second N-terminal domain (α2) holds 10 cysteines, including a Cys–Cys double motif. The C-terminal extension holds five Cys residues, including again a double cysteine motif (Figure 3). Compared with the novel three-domain structure of an MT recently published for *Littorina littorea* [36] and primary structure data of the CdMT of *Helix pomatia* [12,16], it appears that the protein of *Biomphalaria glabrata* may be the first described three-domain MT of a gastropod species consisting, apart from the C-terminal extension, of three highly homologous α domains. In mammalian MT-2, two types of metal-binding domains can be distinguished (designated as α and β) that differ in their number of contained cysteine residues and consequently, in their metal binding stoichiometry [37]. Accordingly, the MT-2 α-domain contains eleven to twelve cysteine residues binding four divalent metal ions, and the β-domain includes nine cysteine residues binding three divalent metal ions.

While the mammalian (N-terminal) and the gastropod (C-terminal) MT β domains share a high degree of similarity [36], less similarity is found between the α domains of *Biomphalaria glabrata* MT and mammalian MT-2 (yielding, for very short sequence stretches, identities of 38–44% and expect values between 2.6 and 8.4). The *MT* gene of *Biomphalaria glabrata* achieved its extended length, apart from a cysteine containing C-terminal extension, presumably by modular triplication of an ancestral α-domain. As observed in a recent study about MT evolution in bivalves, various MT isoforms of the oyster, *Crassostrea virginica*, may have achieved their multi-domain structure by a series of exon and gene duplication events [38]. Upon comparison of protein and gene structures of the *MTs* from *Biomphalaria glabrata* and *Helix pomatia* (Figure 3), it appears that, at least partially, this hypothesis may also apply to the MT of *Biomphalaria glabrata*. The first two domains of this MT align nicely with the exon/intron structure of the gene (Figure 3) and suggest, exon-specific domain duplication. In contrast, the third α-domain does not reflect this hypothesis and may better be explained as a result of protein-specific domain duplication. No indications for exon-specific domain duplications were found in the *CdMT* gene structure of *Helix pomatia* (Figure 3). Hence, the domain structures of both MTs cannot be explained by exon duplication alone.

### 2.4. Allelic Variations

**Figure 4:**
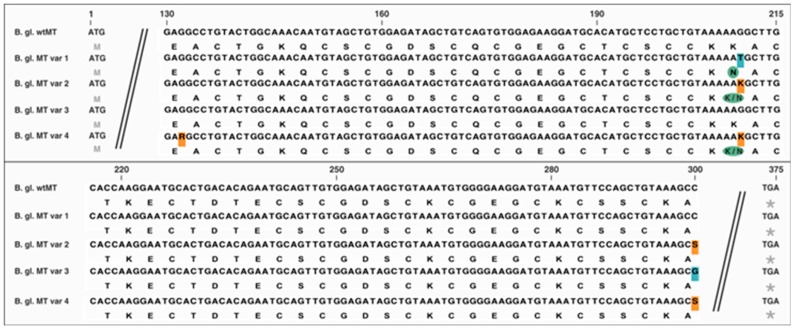
Alignment of the *Biomphalaria glabrata* wild-type MT (B. gl. wtMT) and its four allelic variants (B. gl. MT var 1–4). Parts of the sequence showing 100% coverage amongst all MTs were cut (two black lines). Nucleotide positions are indexed above the top sequence. Amino acids are displayed in single letter code underneath the respective base triplets and the start and stop codons are marked in grey. Nucleotide mutations are shaded in blue, single nucleotide polymorphisms (SNPs) are shaded in orange and the respective changes of amino acids are indicated by a green circle or ellipses. B. gl. MT var 1 shows a change at position 210 (G is replaced by T—changing the codon from lysine to asparagine). B. gl. MT var 2 shows SNPs at positions 210 (K: represents G or T-changing the codon to either lysine or asparagine) and 300 (S: represents G or C). B. gl. MT var 3 shows a shift at position 300 (C is replaced by G). B. gl. MT var 4 shows SNPs at positions 133 (R: represents A or G), 210 (K: represents G or T—changing the codon to either lysine or asparagine) and 300 (S: represents G or C).

The coding region of the *Biomphalaria glabrata* MT shows a considerable degree of allelic variation caused by single nucleotide polymorphisms (SNPs) or mutations of single nucleotides. A screening of MT cDNA sequences from 20 individuals revealed the existence of four allelic variants that differed from the wild-type MT at three different nucleotide positions (133, 210 and 300) of the coding region (Figure 4). MT variant 1 shows a nucleotide mutation at position 210, changing the respective amino acid from lysine to asparagine. MT variant 2 shows SNPs at nucleotide positions 210 and 300, changing the respective amino acid position to either lysine or asparagine in the former, and the latter being silent. MT variant 3 differs from the wild-type MT due to a silent nucleotide mutation at position 300, and MT variant 4 shows two SNPs at nucleotide positions 210 and 300, identical to those in the MT variant 2, and beyond that a silent SNP at nucleotide position 133 (Figure 4). Since in many gastropod MTs, there is a clear relationship between primary structure and metal binding specificity, we assume that the variants of *Biomphalaria glabrata* MT may slightly differ with respect to their metal binding behavior [35].

### 2.5. Lacking Upregulation of the MT Gene Due to Cd Exposure

**Figure 5:**
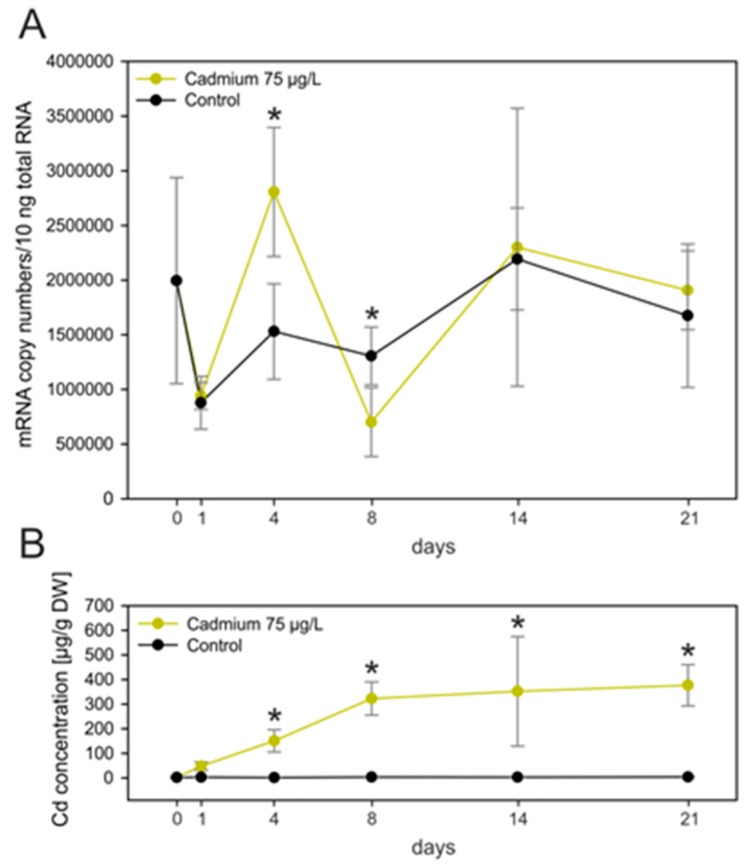
(**A**) *MT* induction pattern in the midgut gland of untreated individuals (black line) and individuals exposed to cadmium (75 µg/L) (yellow line) of *Biomphalaria glabrata* over a period of 21 days. Means and standard deviations are shown (*n* = 4). The course of MT mRNA transcription was tested by two-way analysis of variance (ANOVA) (*p* ≤ 0.05) but was insignificant. Stars indicate significant differences at single time points according to multiple *t*-test comparisons; (**B**) cadmium concentration in the midgut gland of *Biomphalaria glabrata* from controls (black line) and exposed individuals (75 µg/L Cd) (yellow line). Means and standard deviations are shown (*n* = 4). Two-way ANOVA (*p* ≤ 0.05) revealed significant differences between the two treatments. Stars indicate significant differences at single time points according to multiple *t*-test comparisons.

In contrast to several metal-specific MTs from other gastropod species [14,32,39,40,41], the *MT* gene in the midgut gland of *Biomphalaria glabrata* did not show significant transcriptional upregulation upon exposure to sublethal concentrations of Cd^2+^ (75 µg/L) (Figure 5A), in spite of the strong metal accumulation in this organ during exposure (Figure 5B), and no observed mortality in metal-exposed snails. Instead, it appeared that the constitutive expression of this gene was already highly elevated under control conditions, with values ranging from ~750,000 to ~2,000,000 copies/10 ng total RNA in unexposed individuals. Even the highly fluctuating transcription levels of MT mRNA during the first 8 days of exposure (with single values significantly above and below control levels on days 4 and 8, respectively) do not vary much the impression that the long-running trend of MT mRNA induction through the whole exposure period remains unaffected by Cd exposure (Figure 5A). In fact, the analysis of variance (ANOVA) test for the time-dependent mRNA expression curve of the Cd exposure group failed to indicate any significance and overall, the fluctuating time pattern of upregulation does not seem to reflect a typical induction pattern as observed for *MT* genes of other gastropod species [14,39,42]. 

As shown in Figure 5B, Cd accumulation in the midgut gland of *Biomphalaria glabrata* seems to reach some kind of saturation after Day 8 of the experiment, although the exposure was still persisting. This suggests that during this time, metal accumulation may go hand in hand with some degree of elimination, keeping the accumulation curve at a constant equilibrium. Unfortunately, we do not know the mechanisms of elimination in this case. However, it could be suggested that they may in part consist of cellular mechanisms of excretion, as observed in other gastropod species [43,44,45]. Overall, it can be concluded that despite Cd being accumulated strongly in the midgut gland tissue of metal-exposed snails (Figure 5B) with a concentration factor about 400 times above control levels, no concomitant upregulation of the MT mRNA could be observed. This might be because the constitutive expression of this gene was already high enough to inactivate Cd^2+^ ions entering the midgut gland cells. Since the MT of *Biomphalaria glabrata* is not Cd-specific at all [35], metal binding to the protein may in this case be achieved through exchange reactions at the protein’s binding sites, for example with Cd^2+^ ions replacing Zn^2+^. This also suggests that MT may not necessarily be the main actor for Cd detoxification in *Biomphalaria glabrata*. Alternatively or concomitantly, phytochelatines may be involved in Cd detoxification, as shown for Cd-exposed *Lymnaea stagnalis*, a close relative of *Biomphalaria glabrata* [46]. Moreover, diurnal variability of physiological activity and age-dependent *MT* gene expression, as shown for *MT* genes of the land snails *Cantareus aspersus* and *Helix pomatia*, may also play a role [32,47]. Interestingly, strains of *Biomphalaria glabrata* susceptible to infestation by the parasite *Schistosoma mansonii* have been shown to be more Cd-tolerant compared to parasite-resistant strains [48]. It is not known if the laboratory population of *Biomphalaria glabrata* used in the present study may represent a parasite-susceptible strain.

### 2.6. Transcriptional Response of the CdMT Gene Following Heat and Cold Shock Exposure

**Figure 6:**
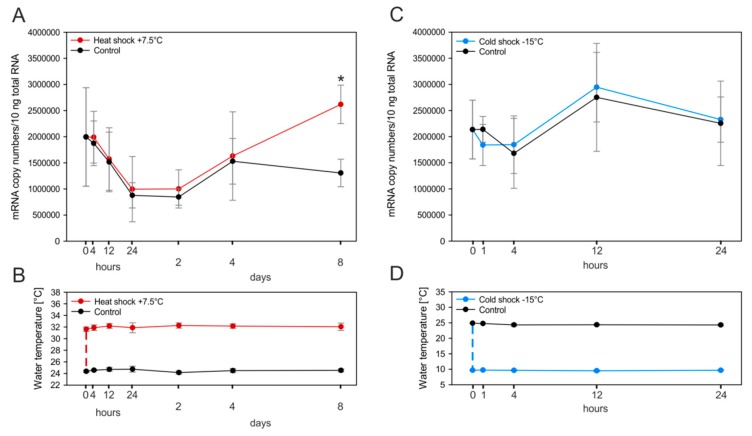
(**A**) *MT* induction and pattern in the midgut gland of *Biomphalaria glabrata* in untreated individuals (black line) and snails exposed to a sudden temperature increase (+7.5 °C) (red line), persisting through a period of 8 days. Means and standard deviations are shown (*n* = 4). The course of MT mRNA transcription levels was tested by two-way ANOVA (*p* ≤ 0.05) but was insignificant. The star indicates a significant difference at a single time point according to multiple *t*-test comparisons; (**B**) water temperature profile for the duration of exposure. The dotted line indicates the heat shock at day 0; (**C**) *MT* induction pattern in the midgut gland of *Biomphalaria glabrata* in untreated individuals (black line) and animals exposed to a sudden temperature decrease (−15 °C) (blue line), through a period of 24 h. Means and standard deviations are shown (*n* = 4). The course of MT mRNA transcription levels was tested by two-way ANOVA (*p* ≤ 0.05) but was insignificant. (**D**) water temperature profile for the duration of exposure. The dotted line indicates the cold shock at day 0.

The presence of multiple heat shock elements (HSEs) as potential binding sites of heat shock factors within the promoter region (see Figure 2) suggested the possibility that the *MT* gene of *Biomphalaria glabrata* may respond to temperature-related stressors, e.g., heat or cold shock exposure (Figure 6) [49]. The ability of MTs to respond to both decreasing and increasing temperatures of exposure has already been reported [50,51] although the distinct mechanism remains unclear. Heat shock with an abrupt increase of the temperature by +7.5 °C applied through 8 days (Figure 6A,B) did not lead to a sudden response of the *MT* gene. Instead, a significant increase of *MT* upregulation could only be observed on day 8 of exposure (Figure 6A). The biological significance of this finding remains unclear, since mRNA upregulation of heat shock-responsive genes would normally occur much faster than in the present case [52].

In the same manner as sudden heat increase may be able to induce genes involved in stress response, a cold shock might as well be an inducer of these genes [49]. In our study, a temperature reduction by −15 °C (Figure 6C) through 24 h did not alter *MT* gene expression levels (Figure 6D). Rather, the mRNA copy numbers of snails subjected to the decreased temperature varied only slightly when compared to control individuals.

## 3. Materials and Methods

### 3.1. Animals, Rearing Conditions and Experimental Set-Up

Individuals of *Biomphalaria glabrata* originated from a laboratory-grown culture at the Institute of Zoology in Innsbruck, where the snails were kept in freshwater aquarium tanks at 25 °C with a 12:12 h photoperiod. Snails were fed *ad libitum* with commercially available lettuce (*Lactuca sativa*) every third day. The trial was approved by the austrian science foundation (Fonds zur Förderung der wissenschaftlichen Forschung (FWF) (Project ref. I 1482-N28; 1 Jan 2014).

Prior to experiments, individuals of *Biomphalaria glabrata* were acclimatized for two weeks in reconstituted water (KCl 18 mg/L, MgSO_4_ 190 mg/L, NaHCO_3_ 98.5 mg/L, CaCl_2_ 450 mg/L and NaCl 430 mg/L in milliQ water) at 25 °C and subsequently separated into different tanks. Three different exposure regimes were applied.

1. **Cadmium exposure:** 40 individuals were subjected to a nominal Cd concentration of 75 µg/L by adding CdCl_2_ to the water. According to our own experiments and data from the literature, Cd LC-50 values at 96 h for *Biomphalaria glabrata* range between 0.1 mg/L [53] and 0.3 mg/L [54]. Forty control snails were kept in Cd-free water. Measured Cd concentrations in the water tanks were as follows (mean ± standard deviation, *n* = 5): Control, 0.24 ± 0.14 µg/L; Cd exposure, 63 ± 7.6 µg/L. Four snails of each group were sampled on days 1, 4, 8, 14 and 21.
2. **Heat shock experiment:** Post-acclimatization, 40 snails were directly subjected to tanks with an increased water temperature by 7.5 °C to exert a heat shock. Due to the expectation of a fast response, sampling occurred at 4 h, 12 h and at day 1. For detection of possible long-term effects, sampling was additionally extended to days 2, 4 and 8. Forty snails were kept in 25 °C water as a control and were sampled on day 0 and all other respective time points.
3. **Cold shock experiment:** Post-acclimatization, 35 snails were directly subjected to tanks with a water temperature decreased by 15 °C to exert a cold shock. Sampling was done at 1, 4, 12 and 24 h. 35 snails were kept in 25 °C water as a control and were sampled on day 0 and all other respective time points.

Throughout the experiment, the snails were fed with lettuce (*Lactuca sativa*) *ad libitum*. All sampled individuals were dissected and the midgut gland tissue was used for RNA isolation and tissue Cd analysis as described below.

### 3.2. Primary Structure of the Biomphalaria glabrata MT and Its Variants

The primary MT structure was identified by genome analysis using VectorBase in agreement with main proponents of the *Biomphalaria glabrata* genome project [55]. The complete gene structure was annotated and submitted to the national center for biotechnology information (NCBI) (GenBank acc. nr.: XM_013225031) (Supplementary Material). The respective mRNA sequence (KT697617) (including the 5′ and 3′ untranslated regions) was experimentally verified by sequencing rapid amplification of cDNA ends polymerase chain reaction (RACE-PCR)-amplified and cloned individuals as described below. 20 individuals of *Biomphalaria glabrata* were screened for allelic variation by sequencing PCR–amplified and cloned individuals, revealing four distinct allelic variants (*n* = 3). The respective sequences were submitted to GenBank and are accessible under the acc. nrs.: KY963493 (allelic variant 1), KY963494 (allelic variant 2), KY963495 (allelic variant 3) and KY963496 (allelic variant 4). Putative MT isoform sequences of *Biomphalaria glabrata* published earlier [14] could not be confirmed by genome analysis or PCR. They may have originated from cross-contamination during sample preparation. The respective sequences and accession numbers were therefore deleted from GenBank in agreement with the GenBank support team.

### 3.3. mRNA Isolation, Reverse Transcription, RACE-PCR and Sequencing

Total RNA was isolated from homogenized (Precellys, Bertin Instruments, Montigny-le-Bretonneux, France) midgut gland tissues of freshly dissected, untreated Ramshorn snails (*Biomphalaria glabrata*) originating from our laboratory culture at the University of Innsbruck, using the RNeasy Plant Mini Kit (QIAGEN, Venlo, The Netherlands) followed by DNase 1 digestion (Invitrogen, Thermo Fisher Scientific, Waltham, MA, USA). cDNA libraries of 23 individuals were generated using the Moloney murine leukemia virus reverse transcriptase (M-MLV RT) reverse transcriptase (Invitrogen, Thermo Fisher Scientific, Waltham, MA, USA). Three of these individuals were subjected to rapid amplification of cDNA ends (RACE-PCR) and the other 20 individuals were screened for allelic variation with the Titanium Taq PCR system using the following primers (located in the 5′ and 3′ untranslated regions): forward 5′-AAACACCATGAGTGGCAA-3′ and reverse 5′-CCACTCAACTCTTACAGC-3′ according to the recommended protocol, with a denaturation cycle of 95 °C for 1 min, followed by 30 cycles with 95 °C for 30 s, 50 °C for 50 s and 68 °C for 50 s, with a final extension step at 68 °C for an additional 3 min. RACE-PCR (*n* = 3) was applied using the SMARTer RACE 5′/3′ Kit (Takara Clontech, Shimogyo-ku, Kyoto, Japan) with the following primers (GSP1: 5′-ACTGAGGCTTGTACTGGGGA-3′, GSP2: 5′-TTGCATCCCTCTCCACATTTAC-3′) according to the recommended protocol. Amplified products were separated by a 1.5% agarose gel, stained with GelRed (Biotium Inc., Bay Area, CA, USA) and subsequently purified using the Qiaquick Gel Extraction Kit (QIAGEN, Venlo, The Netherlands). Cloning of PCR fragments was performed with TOPO TA Cloning Kit for Sequencing (Invitrogen, Thermo Fisher Scientific, Waltham, MA, USA). Plasmids were purified with the QIAprep Mini-Prep Kit (QIAGEN, Venlo, The Netherlands) and sequenced using the BigDye Terminator version 1.1 Cycle Sequencing Kit (Applied Biosystems, Thermo Fisher Scientific, Waltham, MA, USA). DNA Sequencing Analysis Software v5.2 (Applied Biosystems, Thermo Fisher Scientific, Waltham, MA, USA), CLC Workbench software (CLC Bio-Qiagen, Aarhus, Denmark) and ClustalW [56] were applied for sequence analysis.

### 3.4. Bioinformatic Analysis

Analysis of putative transcription factor binding sites (TFBs) for the *MT* gene of *Biomphalaria glabrata* was performed by means of the transcription factor database (TRANSFAC) software package (version 2014.4, BIOBASE, Wolfenbuettel, Germany) [57]. The program was run with default parameters except for the “matrix similarity” setting, where the parameter was set to 0.9.

### 3.5. mRNA Isolation, Reverse Transcription and Quantitative Real-Time Detection PCR

Individuals were dissected on an ice-cooled stainless steel plate and total RNA was isolated from ~10 mg of homogenized (Precellys, Bertin Instruments, Montigny-le-Bretonneux, France) midgut gland tissue with the RNeasy^®^Plant Mini Kit (QIAGEN, Venlo, The Netherlands) applying on-column DNase 1 digestion (QIAGEN, Venlo, The Netherlands). RNA was screened for integrity visually on an agarose gel and quantified with the RiboGreen^®^RNA Quantification Kit from Molecular Probes (Invitrogen, Thermo Fisher Scientific, Waltham, MA, USA) on a VICTOR™X4 2030 Multilabel Reader (PerkinElmer, Waltham, MA, USA). First strand cDNA was synthesized from 250 ng of total RNA with the Superscript^®^ IV Reverse Transcriptase synthesis kit (Invitrogen, Thermo Fisher Scientific, Waltham, MA, USA) in a 20 µL approach for subsequent real-time detection PCR. The remaining tissue was processed further for Cd analysis as described below.

Quantitative real-time detection PCR of BglMT cDNA was performed with Power SYBR Green (Applied Biosystems, Thermo Fisher Scientific, Waltham, MA, USA) on a QuantStudio 3 (Applied Biosystems, Thermo Fisher Scientific, Waltham, MA, USA). The transcript with the defined amplicon length of 107 bp was amplified using the following concentrations and primers: sense primer: 900 nM; 5′-GCACTGACACAGAATGCAGTTG-3′ and antisense primer, 900 nM; 5′-TTTGCACCCTTCATCTGACTTAGT-3′ applying the following protocol of 40 cycles: denaturation at 95 °C for 15 s, annealing and extension combined at 60 °C for 60 s. The 10 µL PCR reaction contained 1 µL of cDNA and 1× Power SYBR Green PCR master mix, 1× U-BSA and sense and antisense primer. Primers were designed using the Primer Express 3.0 software (Applied Biosystems, Thermo Fisher Scientific, Waltham, MA, USA) and a primer matrix with subsequent dissociation curves was used to determine optimal primer concentrations. Calibration curves from amplicons were generated to determine *C*_q_ values for copy number analysis (PCR efficiency ~96%) using the Thermo Fisher Cloud Software, Version 1.0 (Life Technologies Corporation, Carlsbad, CA, USA).

### 3.6. Metal Analysis

Cd concentrations in the midgut gland tissues and the medium were assessed by means of atomic absorption spectrophotometry. After oven-drying of tissue aliquots at 65 °C and dry weight determination, samples were digested under pressure in 2 mL tubes (Eppendorf, Hamburg, Germany) with a 1:1 mixture of nitric acid (65%) (Suprapur, Merck, Darmstadt, Germany) and deionized water in an aluminum oven at 69 °C. After obtaining a clear solution, the samples were diluted with deionized water to 2 mL. Subsequently, Cd concentrations were measured with an atomic absorption spectrophotometer (model Z-8200, Hitachi, Tokyo, Japan). The system was calibrated with standard Cd solutions in 1% nitric acid and the accuracy of metal measurements of the midgut gland was verified using certified standard reference material (TORT-2, Lobster Hepatopancreas Reference Material for Trace Metals; National Research Council Canada, Ottawa, ON, Canada).

### 3.7. Statistical Methods

Data of qRT PCR and metal analysis were evaluated statistically by means of SigmaPlot 12.5. For normal-distributed data, the *t*-test was applied whereas for data failing equal distribution the Holm–Sidak method was used. Statistical significance was set at *p* ≤ 0.05. Additionally, analysis of variance (ANOVA) was applied to test for significance of time-dependent variations of data (*p* ≤ 0.001).

## 4. Conclusions

Our results show that *Biomphalaria glabrata* is apparently highly tolerant to Cd^2+^, as demonstrated by the high metal concentrations accumulated in the snail’s midgut gland. In spite of this, there is no upregulation of the *MT* gene due to Cd^2+^ exposure. Moreover, there is no increase of MT mRNA due to application of temperature shock exposures (heat and cold). It is assumed that the high constitutive expression of this MT may counteract metal stress by unspecific binding through metal exchange reactions at the protein’s binding sites [35]; in a similar way, the high constitutive expression level may also contribute to heat and cold shock tolerance. Alternatively, and/or concomitantly, Cd detoxification in *Biomphalaria glabrata* may also be achieved through complexation by phytochelatins [46].

## Figures and Tables

**Table 1:** Theoretical binding sites for transcription factors involved in stress-response and transcriptional regulation from the promoter region of *Biomphalaria glabrata*. Abbreviated terms indicate the binding sites and suggested functional context according to explanations in TRANSFAC databases (see materials and methods).

**Response Elements and Binding Sites for Transcription Factors Involved in Stress Response**		
Response element or binding site (abbreviation)	Explanation	Functional context
C/EBP	C/Enhancer binding protein	Glucocorticoid activation
HSE	Heat Shock element	Heat shock protein activation
MRE	Metal responsive element	Metal-induced expression
PPAR	Peroxisome proliferator activated receptor	Stress response
YAP	Yeast activator protein	Multidrug resistance in yeast [34]
**Response Elements and Binding Sites for Transcription Factors Involved in Transcriptional Regulation**		
Response element or binding site (abbreviation)	Explanation	Functional context
AP-1	Activator Protein 1	Nuclear transcriptional activator
ATF	Activating transcription factor	Transcriptional activator
cMyc	c-myc protein	Telomerase activator
GATA-1	GATA binding protein 1	Cell growth transcription factor
POU1F1a, POU2F1	Octamer-binding factors	Transcriptional activators
p300	E1A-associated 300-kDa protein	Transcriptional enhancer
PBF	Prolamin box binding factor	Transcriptional activator in plants
TBP	TATA-binding protein	Polymerase activator
Cdx-1	Cdx homeodomain protein	Activator
MEF-2C	MADS box transcription enhancer factor 2	Transcriptional enhancer

## Supplementary Materials

Supplementary materials can be found at www.mdpi.com/1422-0067/18/8/1747/s1.
