# Supplementary material for: Challenging the Metallothionein (MT) Gene of Biomphalaria glabrata: Unexpected Response Patterns Due to Cadmium Exposure and Temperature Stress
Source: Int J Mol Sci. 2017 Aug 11;18(8):1747. doi: 10.3390/ijms18081747 (PMC5578137; doi:10.3390/ijms18081747)
Supplement: Supplementary file 1 [file ijms-18-01747-s001.pdf]

Supplementary material:

Hypothetical MT gene of Biomphalaria glabrata:

The MT gene of *Biomphalaria glabrata* was identified and annotated within the *Biomphalaria glabrata* Genome Project and is publically available at VectorBase (available online: www.vectorbase.org). It has been released to GenBank (acc.nr.: XM_013225031.1; Gene ID: 106066062).

gtatttttagttcaacacatggcacagtctcatttataaatacgattatgtatatatgtttcaaatatcaataagtattattttatttaaattaataact
aattattcatttatgtcttttttttctcataaaagtgcgtctacgggagggatacgattgggtgatcacacctaccgcccttccaggattagtttgaat
ggccctgccaaaaccaatcaaaatatctgaaagaaacaaactaacacgtagggctcttcaatttagggaaataacaaaagaatggaaagaagc
atttcatctcactgtttaaagttggtagcattaaaaatttttaaatacattttgataataatatcctttagccacatttttccccaaactatctcctatg
acaaggtcaacttggttatcttttcatcacttttatcagctgaatctcgtgagtgatgagtcacatagaagtccaagtggagggaaaaaagccaca
attagagaagatcattgaactaaaaagtatgtgagactgagacctccaagaagccagaataacgtcacagaaccgtcagaaacacgtcaaatt
aaaagaataaataattttgttgtagaattttgagctatatttccctaaccaaaactaaaggtcatagtaaccgtagtttgactgaaatcactcgtag
atctacgttacggtagttcacttattgtgactataggctgaggagtctgtgtgtgggtaatgtaattcttgatggaccatgtggtgtttttttcttcata
tctgttgtgttgtatttgcatttttaactaatgcgtcaagtaatctcacaatttcaaaataaaataaaacgcctttttttctcttttgtttacgatgtagt
aaatctatttctaaattagtaatcttggtcaacagaaatgaaacaattctaaacacctgatcatttataagaaaagaaaattcaactcgttttgaca
cgctctacaataaaattcgatgcacttttaacttatcgatttttttgagtgatgcccagtctatttatagtaacaaggacagtaaacgagccattgca
aatgctaatgacaagtgacaagggttaatctagtattatactgttgtagctagtgacagaactgtttgtcttacagttgtttaaaaaaaggtttattt
ttctaaagaacaataaaatacgtagttctaaacttcaaatctatttagccacatgggttttcttttatgtccatgaaagtacaacggacatctggat
ggccagtgttgagacgacaatccgtgtgcaaatttatcgcatggctgttagaatgatctcgattttggccgtgtgcatataacggccttggttggca
agtaggctatataaaccagagactgtctactttctctattgaattcctcgttgatcaagaatattttattttaacagttcataacaacaaacaccatg
agtggcaaaggtaagaagaggaatcattatttccctttgtttgaaaaaatgacgcatcgtaaatgtcttaggaactataacgtgtttttgagtaga
ctgactgtctactattataacaatagatcttgacattgtagctgcttaggcttttatatagcagaccacacatttaaggctaaattgttgctaagatg
atgtgatttgttttacacattataaatttagtttgtcaaattaactaacactgactgctagaagacaactaaataatgaatccaattataaaataaa
taaatagtttattagagttaaaactttacttaagtaaaaaaaaaaggatgactaacaaaatattgaaatattgatttattttttgatactacatgtt
acagttatataggctactaccatgaactgtagagcatatatttatatatcatagtgcaggctatacatgatagataaaggctaggccgttaggcta
taaatgctaagctattgctacacttttattctatattttttttaatatgatgtctctttttgtttttttccaaaattttaacaggtccctataaatttacaag
gcataagcttaccctgattccaaatatgcataaggcatggtatgatggttgtatgttgaacacgtcataataagaaataaccaagataaataaaa
gaagtgtgtacgtgcataatggggtataggctactgagatgggggggggcatacagctatacataatcgcggcttcttccatacgacaaccatt
aaaacataagcctattagtaaaccaaggttttactatagcctacttttgcttgtatgccgtggaaaccatttttttttattactaatgttaaaataaata
aagaagacgggaaggtatcgaaacatttgcatatcagtaaatcatttttttaaatctttgcaaacattaataattattagttcaagtttctaaagctt
aaataaacttgtattgaatgagaaaatataaagtgcccaataaacctataggtcgctgggactattgtaaattggaatgactaaactgatgccgc
cactaggattgccactgggacacttaaaataaatgtgactgatgatgaaaagctcttctattttgtctatttaatttctttctagtcgacaaacacaa
cgaccaaaaagtcttagatatagatatctaataaaactgaatgtcgttaggatatatagatatctaataaaactgaatgtcgttaggatatataga
tatctaataaaactgaatgtcgttaggaatatctacatttcaaattcctcattttcgtggtagtttggtcttgtacaaatccgccatctagccaactgt
ctcttagtaggccctacatctaactattacaccgtcttatgttcaactataagattaccggtaaagttcgtcactactgagtcagtttttattttgttta
ttcatggagatggcttcgaggcctgtgatgaatagagcattcaatatttggcattgattttgtatcgcacatctttcatgatgtaggctaacttgccc
aatgcgctgaggtccattctcttatctggagattgtcggagggaggagtggtggcgagaaacagtttctatgctgcctttaggcattcagctcaagt
cgggataggtagccaaacggtttttcccactcagccccacatacaaattgacatgcagatttggacatgaaaagctatatgtgtttgatttataata
gaaataaatttgaaatataatataggggcctacttatgtttagagtttagacagacaagctcgagcaaaaaaaaatctgttattattcataccagc
aaatggcagtaatgaaaagatctatccctttcaacaacaacaacaaaaacacgatgaatttttttgtttcaaataatttttttttaaatttcagttaca
aatgtagcgcctctagtcttaaaactaccacctaaaagtttcactatgtgtgttttcatgggtttgtccaaaaaacaaaacaacaaaaacacttcgt
aatcacattattaatttagtttatacaaatagtagacctactgtcaagcacttatttttatcagggatctcacgagcaacagtaaatcaaaatgaaa
tgaaggcaagaacgacagctgactgtagtgttaccaatcaatttttatccaagtctgacaccggccttgtttttcagtcgtattattctaattcttttc
tatgcacatttggacacgtgggatgtcatttaacttaagatatacaaaacattgtttttattttgtaattagatactgccattgtgttacaatgtttatg

aaaactcttttttgaacatatcagcaggaatacgataggacaaaaaaacaaaaagcaagccaaagcacaagccttcaataaccgacatgagac
tcaccagtcacgtagcgtattgttattttacccagatctaaaactcaggctgttttattgatccttataggaaattgtgattacaagggttctttttca
ataaaaaaaacattccccaaaagagaacagacgtgacagacagacaagttcacaggggactaaacatggacatcttgatttgatcttttgtca
cgttcccttcacattgcacgcccaacccacctctttttgattttttggtgtttgcagggttgtattccacagactgtagattttgttcggccttctaacac
ccattatatctcaaaatcatttgctagtgaatgtctgagcttgtctgtattaggtacaactaatacatgtaaatatcattacttgattaatgaaatttt
aatgactggttgagggaaaaaaggactttatttaaaatagtatttagatctaaacttaattcgacatagtaagaaagatgttagttcaagaaaga
tgttagtgctacacaaaatgttacaagcttattaattaccttttaatattgagtgtaattgcctgttgttgttgttttttttttttgttttgtttttttattgat
tcatgcattgtcatcgactatgtacaattcttcaaattttttaattaattcgagaacggaaagtgggagacaaaaagggtatcgtacataacgtaat
aagggggaacactccacgtatacagccacatatctcaacaagtagcatctatttcctttataactttttcgataccaaacaaaatatttatttgtca
ataattaataaactaattggtcaattatttttaattgattttaaatggaaaataatagtgtaaagtttcaatttgatccgagaatgggtgtgggaga
aataacgtgtacaaaagttgtaccagacagacacaaacagagtgagttgataaaaatatataagctttgtcattaaaacaaaaaaattaatggc
agtttaaaagagaaactgagtttgtccaatgcagacaagacagctataattcaaacctaccagtggcttaaatattattatgtttttgtttttcattt
aaaatccagtcatttttgttttcataggcaatgagattgtgtctgaaaagaccttcatttatttattctgtaggcacctcttcccagacatgtcaataa
tacttttaaagaggaggaattaaacagcaaacgcttccgttttaccagtagttgggactaaatatatatttacgatgatttttgaaaaaaataaat
actacattgtttggcctgcttacaaacttatttcgtagaatgtatcttttgagtcgtgctaaatcaatgaaaacgtgtgcaaaatctctgcacttgtag
ataaactatatataagtggagagacgtgcgtccagtaatcatgatatttgtttgtttgtttgtgggtgtgtgtgtatatggaaagggggagtggaat
aaaaggacaatgccgtgtgcaaaccataaactttggcagaaaaaaactctaaataaatatatgactttgttttgtgaaatgataagtacgctccc
cttttgctctacttttataataactttttttttttaaaaaaaaggtcattaaaagttcatctttgtctagttgtagcctaattcaagtctatagatagcccg
agttatcatgttattgtgatattttgtagcccatggtatgaccgaccaataaaagtgggtgtgagtttttatctctaagataacttctagaatgcttgg
ccttcgtggtaagctcaggcctactaattggcctaccttgagagataattagataaactaaataactcgcttacttgaacgtacacgaacgaaaag
cgctggcctatcttacatctcattggcatattaatttaacttactaagatcatgtacatccacttcaatctccattcttagtatgcgacttggttttcga
aaaaggttatcactctttgcttcaccgatgacaagaatcgatgtctggtggtgataaatagcacgccctttccaggttccgccactctttagccttca
tgtcgatcttatgagttaatcaactgaaataaaatatcttacgtttaatgtctaataggtctactaaagatgctctttaaattcttcttccatgtcaaa
tttcgctggcctcgtttttgaatctattatgttaattacgcagaaggtatctctctctctctctctctctctctctctctctatatatctctatatata
tatatatatatatatannnnnnnnnnnnnnnnnnnnnnnnnnnnnnnnnnnnnnnnnnnnnnnnnnnnnnnnnnnnnnnnnnnnnn
nnnnnnnnnnnnntactatatatatatatatatatatatatatatatattatattatacttatactactagtactatatatatatatatatatatat
atatatatacataccggtatatagatttattattctcgtcatggctcaagagtttggacaaggagtaaaggaaagatcattcttttatttctgcaagt
cggactagagttaccctacgaaaaaaaaagggggggggaagagaacaagtagtattcaccacaaaatagcagggccggactgaaccattgtg
ggcccaatgcgaaatggatttcgcggggcccagtttgggtaggaatacggataataagtgaaaattaaaagtttgtattagaaaatatattcgtct
ttccattttattcattctttactacgcacagaaaaactacacgagcgaagtcatacattaggcggctgtaggactatataataagaattcagtttcct
acatagatcacgctcaatagcaagaattaccaaatgtttcaatctattttagagaattgttgacctcaagtaattcttcgttagtttgagacgcgag
aagcttctttcagtagattccacaattacgggtattgcattatgccgctttttcttttatcgcgcgtaggattggcgttttccatattgaatgacacccc
aaaatgacaatttttgtatatatatttcaggagatatgtatgagttttttaaagattttaataatttacggatatttccatgcctttttcgtatattttgc
actttcaggagatttccaggagctcctggtaaatcgacaggaggccgtgggaaatttgttataagttataaaatggtttaatttaataatttataca
tctagaattagcgcgggtcctatgaacctgtggggcccattgcggttgcatagattgccctgcaaaatagcggcgtatgctccgccgccggtcgac
tagttctgtatattagaaatgtcaaacatgagagttatgttaaagcctcatctggaataatggctgtaggaacgtactgtattcctaagattaacag
agccgtagacataggctataagcctacgctacatgttattctaatgttctcacacatcaacatcggcttaacaacacatttttttcattaacagtagg
cctatgcctaatcgaatactaaaagtgcgagatcaaatagtgtgttgttttgttttataacaagacacgaccaataaattaatctccacaggtgtaa
tattaataaataaataaaagtaatgttttaaagtttttccaattgtctaatgctgtgtttaacttagtttaaataataagattcataggccaacgtaa
aatggaagtaatgcgttatagataggttaataatggaacattattaagcaaagtagtagagtagtagtagcatattattgtgtgcgttataattgtc
catgatttatattttcttataaaaagagtcacgcgaaccagggccggtcctacagattgcgcggggcccattctgatttgtgataaggataagtga
aaattaagatttagtccatatttaatggcaccccgaaatgacaattttgtttctttgtaaggagatttgcatcagttttcagaatatttgaataatttc
aagagattttcatgactttttcgtatatttcgcaagttaaaatggtttaatttaatgatttacacctagaattcgcgcggggcctatgaaagtgcggg
gcccactgcggccgcataggttgcaatggcctaagaccggccctgacacgaactgtcatgattgatactcgcctaacctttcattgatgatctttta
atatagattttacagtgtaatattaacataatacattgttcttaaggatggaatggtctaacaaaaatataatacgttacgaactaataccaagtgt
agtgtataataccgcaaggtccagagctgaaaatcattactaaattgcattgtacagtcacatataggcctacatgttttgccattcatgcagctgt

taagtttctggcgcattatcaaacttgacagatgtggaaaggtatgaatggatgttcatcatagtacataccatttgtaccccgaattgtaatttatt
gccaggacggattttaaaacccagtcagaccctgctgtacattgtaaaagttaaattactatctacactataaatattttagaagctcttactctca
gagctagtagacctacatatatgtcaatagatctataacattgtgcttctgtttgggggtgggagtttgttagttgttcttttgattcattttgtttttgt
tttggattcagttaacatgtaaagtatgtctccggttgttgttgtttttttaataattaatgagaataacaaacaaatatcttatttaaaatgtagga
ccaaactgtactgaggcttgtactggggaacaatgcaattgcggggacagctgtaaatgtggagagggatgcaactgccccagctgtaaaacta
ctaaaggtgaggggctaattaacgccgctctgaacttatatcagtattaaaccttttctagttaagcaataatgaacttaacaatttttgatgtct
gttaacattcctatgtggtccttttaggtaagtatctcaccccccccaccccaacccaaagggtagtgtaaagtacatgaagagagctcattgat
atttatattgatagaatcaaacgctctgctaacggataattcaattcacaatttctttttcctttactaaattacagaggtattttttaaccttttatt
atgtaatgtaaatagtgtttttataccatgtgttcctatatctatgtgtatttatgtgtgtacatataggacctaactgtactgaggcctgtactggca
aacaatgtagctgtggagatagctgtcagtgtggagaaggatgcacatgctcctgctgtaaaaagggtaaaacgatattttgcttgatttatttatt
gttagatagaatctagacttttaactttatttaatcgagtgatgttttctaagattatcgtacttgaaactcggacaagctataacaagtttttacaa
aaatactgaaaaaaaatgaatgaaaatatctatatgaatgaaattttatccactggggaagactgggatttttaatttcgggatcttcggggcgcc
tctgagtccatctagctctaatgggtacctgacattagttggggaaaagtaaaagcggttggtcgttgtgctggccacatgaaaaccctcgttaac
cataggccacagaaacagatgacctttacatcatctgccctatagacaacaaagtctaaagggggtactttactttactttagataagggagtcca
taaataatgaacgtattgttagatttaaaaaaaactaaaaagaaaaggttttaaaatgtatttaaagaacatctaagtgctatacaatacttttgtt
tttatactaatattttgttttcttaagaaaattgtatttcttttattaaaatgtcaacattactattcttcattggactagcttgcaccaaggaatgcact
gacacagaatgcagttgtggagatagctgtaaatgtggggaaggatgtaaatgttccagctgtaaagccggtaaatgtactaagtcaggtaaac
ttgtgcacctctttataatagtctaaatatgtttactcccagagattagtgctccgagttttaaatatgaactggtaaatatttgtattttattgtgtaa
tatttcttaacaatgtatgttcaaatgtcttccgagacataaggagccatgttcaaatgtgacatttttataaaagactcaaagatagtggatagat
ctaaattacctttttctggaaggggattgacttaactaacgaaaataaaaggaatgtagcaactatatgatctagtttagattaaatctattttcac
aacaaacaaaaaaatgatagtactaaatttgattatctcctttgtaaatgtacgaaattttatgctcatagacttatatattatactagactgtaaacc
ggaaataaattcttatccgcgatttatctattcacagacctatcaatatatatatagatgtttatgtacgtaaactttttttcatagaaaagtaatga
tctttagttttcaaagtttagaaagaaagcaaaattatttctcggatattcacgcaaatatatatgaaacaaagccatttcctgtttgtttccattga
gataatgtttcaaaaatcttagatcgaaataattcatgttgatttaaatcatcttatgtacattaaaatagattgattacctagatctttcttgatgta
tctaaaaaatgcaaaataataattatgagacgagtgtactcttatttttgatgttcaattactagatctagatctaaaaattaattatatgttacatg
ggttagggttgaaaaataaaactttacttcttttaactactttacaaaacaacgccttgatccaactttctaaaacattttcacatttttgtagtgtt
aaaaagtctccatgtccagtctagagtagaaatattctataagtctatggttatgctactaatcacgagcgagttaaattgataaatacagttgtca
atattatttttttaattattatttcagatgaagggtgcaaaaccgaaggtcactgtgccaaagggaaatgctgtaagagttgagtggattaaacta
aactttcccattctactgacctgaaaaacttccaaatgaaactaacttctataatttgattatgtcatgtttaaagttcaatacgtgatctatgtctat
gtacaaatattgtattcccaaaccgtgtaatttaaataaactattatattat

Promoterregion: 1460 bp
Exon 1: 13 bp
Intron 1: 7197 bp
Exon 2: 102 bp
Intron 2: 358 bp
Exon 3: 96 bp
Intron 3: 595 bp
Exon 4: 108 bp
Intron 4: 1015 bp
Exon 5: 56 bp
3’ End: 164 bp

Total length: 11164 bp
